# Heat and chilling stress induce nucleolus morphological changes

**DOI:** 10.1007/s10265-019-01096-9

**Published:** 2019-03-07

**Authors:** Kohma Hayashi, Sachihiro Matsunaga

**Affiliations:** Department of Applied Biological Science Faculty of Science and Technology, Tokyo University of Science, 2641 Yamazaki, Noda, Chiba 278-8510 Japan

**Keywords:** Chilling stress, 5-Ethynyl uridine, Heat stress, Nucleolus, Nucleolus cavity

## Abstract

The nucleolus, where components of the ribosome are constructed, is known to play an important role in various stress responses in animals. However, little is known about the role of the plant nucleolus under environmental stresses such as heat and chilling stress. In this study, we analyzed nucleolus morphology by determining the distribution of newly synthesized rRNAs with an analog of uridine, 5-ethynyl uridine (EU). When EU was incorporated into the root of the *Arabidopsis thaliana*, EU signals were strongly localized in the nucleolus. The results of the short-term incorporation of EU implied that there is no compartmentation among the processes of transcription, processing, and construction of rRNAs. Nevertheless, under heat and chilling stress, EU was not incorporated into the center of the nucleolus. Morphological analyses using whole rRNA staining and differential interference contrast observations revealed speckled and round structures in the center of the nucleolus under heat and chilling stress, respectively.

## Introduction

The nucleolus is the nuclear structure where rRNAs are synthesized and processed and ribonucleoproteins are assembled. Based on electron microscope observations, the nucleolus has three structures; the fibrillar center (FC), the dense fibrillar component (DFC), and the granular component (GC). The DFC region contains rRNA, the FC region contains RNA polymerases and their cofactors such as upstream binding factors, and the GC region is the site where ribonucleoproteins are assembled (as reviewed in Boisvert et al. 2007; Matsunaga et al. 2013; Sirri et al. 2008).

Many studies on animals have revealed that the nucleolus plays an important role in various stress responses (Reviewed in Boulon et al. 2010) such as DNA damage (AI-Baker et al. 2005; Cioce et al. 2006; Kruhlak et al. 2007; Rubbin et al. 2003), heat stress (Carmo-Fonseca et al. 1993; Handwerger et al. 2002; Rubbin et al. 2003), cold stress (Carmo-Fonseca et al. 1993), hypoxia stress (Mekhail et al. 2006; Rubbin et al. 2003), osmotic stress (Cioce et al. 2006), viral infection (Greco 2009; James et al. 2010; Morency et al. 2007; Rebelo et al. 1996) and nutrient stress (Andrade et al. 1993; Hoppe et al. 2009; Mayer et al. 2006; Tanaka et al. 2010). These stresses have been shown to induce morphological changes in the nucleolus and inhibit RNA polymerase activity, resulting in the release of secretion element(s) required for apoptosis or cell cycle arrest. A recent study showed that a secretion from the nucleolus is also involved in tumor suppression (Russo et al. 2017).

Various stresses trigger the p53 pathway to induce cell-cycle arrest, cell senescence, or apoptosis in animals. *Arabidopsis thaliana* (L.) Heynh. has no p53 homolog, but instead has the plant-specific transcription factor family NAC, whose members function in the pathways responsive to various cellular stresses (as reviewed in Ohbayashi and Sugiyama 2018). One NAC transcription factor, ANAC082, is induced under nucleolar stress and is associated with developmental alterations and cell proliferation defects (Ohbayashi et al. 2017).

5-Ethynyl uridine (EU) is an analog of uridine that can be taken up into newly synthesized RNAs. The click-iT reaction enables fluorescent molecules to bind to EU. A similar analog, 5-ethynyl-2′-deoxyuridine (EdU), has been widely used to tag fluorescent molecules to newly synthesized DNA in plants (Hayashi et al. 2013; Ichihashi et al. 2011; Katogany et al. 2010; Salic et al. 2008; Yokoyama et al. 2016). Recently, EU has been reported as a suitable molecule to stain newly synthesized RNAs in *A. thaliana* roots (Dvořáčková et al. 2018). Because rRNA are actively synthesized in the nucleolus (Pontvianne et al. 2013), visualization with EU allows analyses of the morphology and function of the nucleolus. In this study, we analyzed the relationship between environmental stress responses and nucleolar morphology by EU staining.

## Materials and methods

### Plant material and EU incorporation

5-day-old seedlings of *A. thaliana* (Col-0) were used in these experiments. The seedlings were incubated in liquid 1/2 MS (Murashige and Skoog) medium containing 1% (w/v) sucrose and 1 mM EU (Jena Bioscience, Jena, Germany) for 2 h in the dark at the following temperatures: 0 °C, 12 °C, 22 °C, 30 °C, and 37 °C for chilling and cold stresses, control conditions, and mild heat, and severe heat stresses, respectively. For osmotic stress, the liquid medium contained 200 mM NaCl, and for actinomycin D (ActD) treatment, 5, 25, or 60 µM Act D was added to the liquid medium 1 h before or at the same time as EU incorporation. For a time course experiment under heat stress, seedlings were incubated in the liquid medium for 30 and 60 min. After the incubation, seedlings were fixed in 4% paraformaldehyde (PFA) in phosphate-buffered saline (PBS) for 30 min. During fixation, the chambers were evaporated. The fixed seedlings were washed three times with 1 × PBS, then incubated in 0.1% (v/v) TritonX-100 in PBS for 30 min. After washing the seedlings three times with 1 × PBS, EU was detected following the manufacturer’s instructions for Click-iT (Invitrogen, Carlsbad, CA, USA), and stained with 5 µM DAPI (4′,6-diamidino-2-phenylindole; LONZA, Walkersville, ML, USA) for 4 min. After three washes with PBST (1 × PBS, 0.05% w/v Tween20), the samples were mounted with mounting medium (50% (v/v) 2,2′-thiodiethanol (Sigma), 50% (v/v) 1 × PBS, and 2.5% (w/v) n-propyl gallate).

### EU pulse incorporation and release

5-day-old seedlings of *A. thaliana* (Col-0) were used in these experiments. The seedlings were incubated in liquid 1/2 MS medium containing 1% (w/v) sucrose and 1 mM EU (Jena Bioscience) for 2, 5, 15, and 30 min in the dark. To analyze EU release, seedlings were incubated for 30 min in medium containing EU. The seedlings were washed with EU-free medium, after which they were incubated in EU-free medium for 1.5, 2, 3, 4, and 5 h. The EU was detected as described earlier.

### Nucleolar ID incorporation

5-day-old seedlings were subjected to temperature stress treatments as described above, and then incubated in Nucleolar-ID-containing medium (1/2 MS, 1% w/v sucrose, 500-times dilution of Nucleolar ID (Enzo, Farmingdale, NY, USA)) for 30 min. After washing three times with 1 × assay buffer, the seedlings were incubated in 1 × assay buffer for 30 min at 22 °C in the dark.

### Propidium iodide staining

Propidium iodide (PI; Sigma-Aldrich, Saint Louis Missouri, USA) was diluted (1:100) with distilled water for a final concentration of 10 µg mL^− 1^. Seedlings were stained with the PI solution for 2 min in the dark, after which they were examined with a confocal microscope.

### Imaging procedure

The fluorescence of stained samples was detected under a FV1200 confocal laser microscope equipped with a GaAsP detector. Differential interference contrast (DIC) observations were conducted under an upright microscope (BX53, Olympus, Tokyo, Japan) equipped with a DOC camera (Molecular Devices, Sunnyvale, CA, USA).

### Imaging analyses

Images were analyzed with ImageJ (https://imagej.nih.gov/ij/). In the analyses of ActD-treated nuclei, nuclear patterns were classified using the ratio of EU signal intensity. When the average value of EU signal intensity in the nucleolus was stronger than in the nucleoplasm, we defined the nucleolus as S type. When the average value of EU signal intensity in the nucleolus was equal to or lower than in the nucleoplasm, we defined the nucleolus as W type.

## Results and discussion

### EU incorporation was inhibited by ActD

**Fig. 1:**
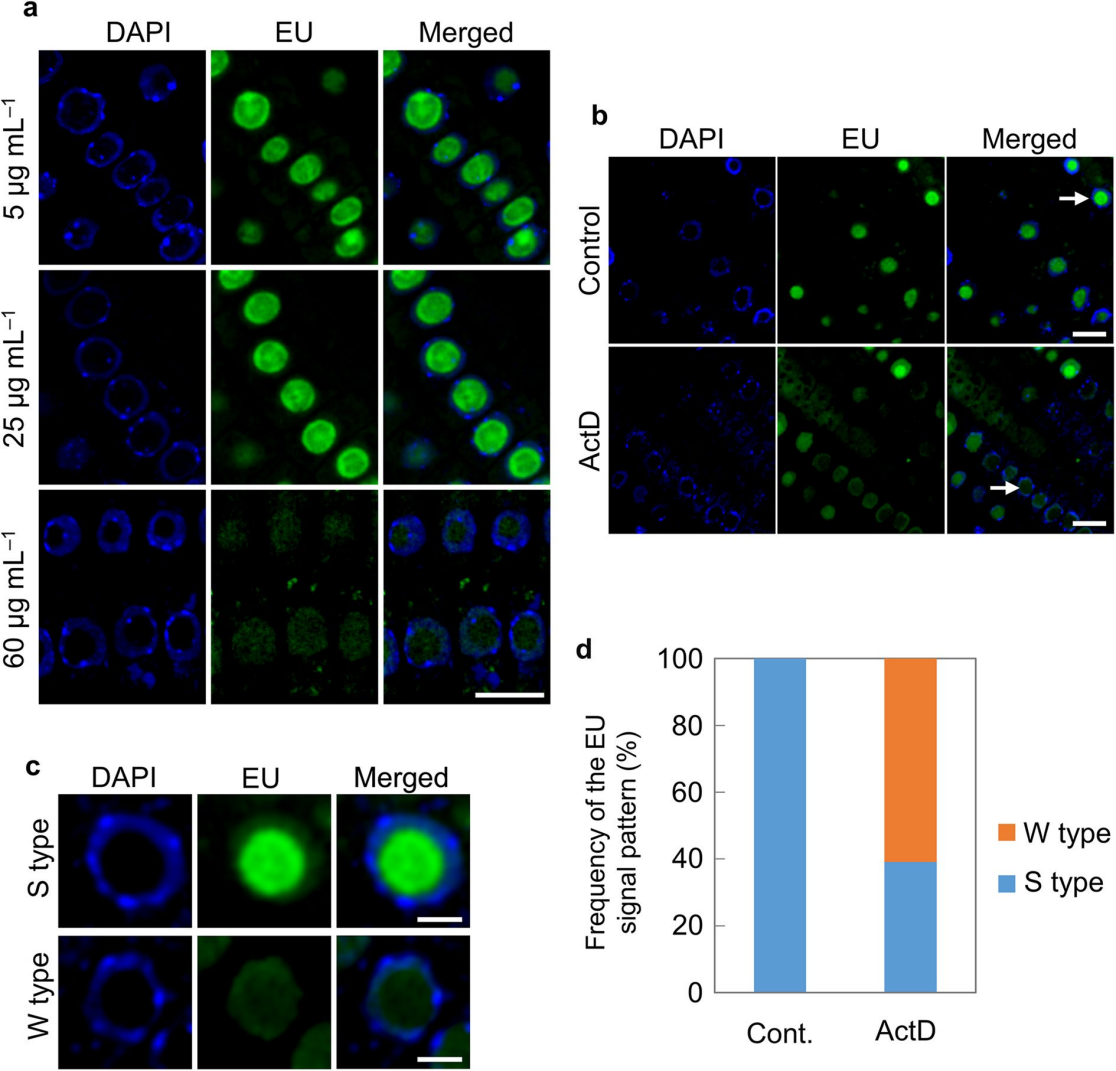
Distribution of 5-ethynyl uridine (EU) in nucleus of *Arabidopsis thaliana* root cells. **a** Root cells of *A. thaliana* with incorporated EU after ActD treatment; 4′,6-diamidino-2-phenylindole (DAPI) staining (left), EU (middle), and merged image (right). Scale bar in the lower right image = 10 µm. **b** Comparison of EU signal patterns without (upper) and with ActD treatment (lower). DAPI staining (left), EU (middle), and merged image (right). Scale bar in the lower right image = 10 µm. **c** EU signal patterns classified as S type (strong) (upper) and W type (weak) (lower). An arrowhead marks the S type in a magnified nucleus from (**b**, upper) and the W type in a magnified nucleus from (**b**, lower). Scale bars = 3 µm. **d** Frequency of S type and W type EU signals without (control) and with ActD (*n* = 69 for the control and *n* = 72 for ActD treatment). The number of the seedlings was 3–5 per experiment

To confirm that the EU signals were derived from newly synthesized rRNA, EU was supplied after 1 h treatment with the rRNA transcription inhibitor ActD, which binds to rDNA and inhibits the binding of RNA polymerase I. ActD concentrations of 5, 25, and 60 µg mL^− 1^ were used with reference to previous experiments (Ahn et al. 2016; Dvořáčková et al. 2018; Mishiba et al. 2013). As shown in Fig. 1a, EU was incorporated into the nucleolus under 5 and 25 µg mL^− 1^ ActD, but was not incorporated under 60 µg mL^− 1^. In Fig. 1b, EU was supplied in combination with 60 µg mL^− 1^ ActD. As shown in Fig. 1b, the signal intensity of EU incorporated into the nucleolus was much weaker when it was supplied with ActD than when it was supplied without. Figure 1c, d shows the frequency of EU signal patterns classified as strong (S type) and weak (W type). In the S type, the intensity of EU signals was stronger in the nucleolus than in the nucleoplasm. In the W type, the intensity of EU signals in the nucleolus was equal to or weaker than that in the nucleoplasm. The predominance of type W in the ActD treatment indicated that inhibition of rRNA transcription by ActD reduced the incorporation of EU into newly synthesized rRNA. However, EU signals were observed in the nucleoplasm. Together, these results confirmed that EU was appropriately incorporated into newly synthesized rRNA.

### EU was incorporated by the whole nucleolus, but was released from the center region

**Fig. 2:**
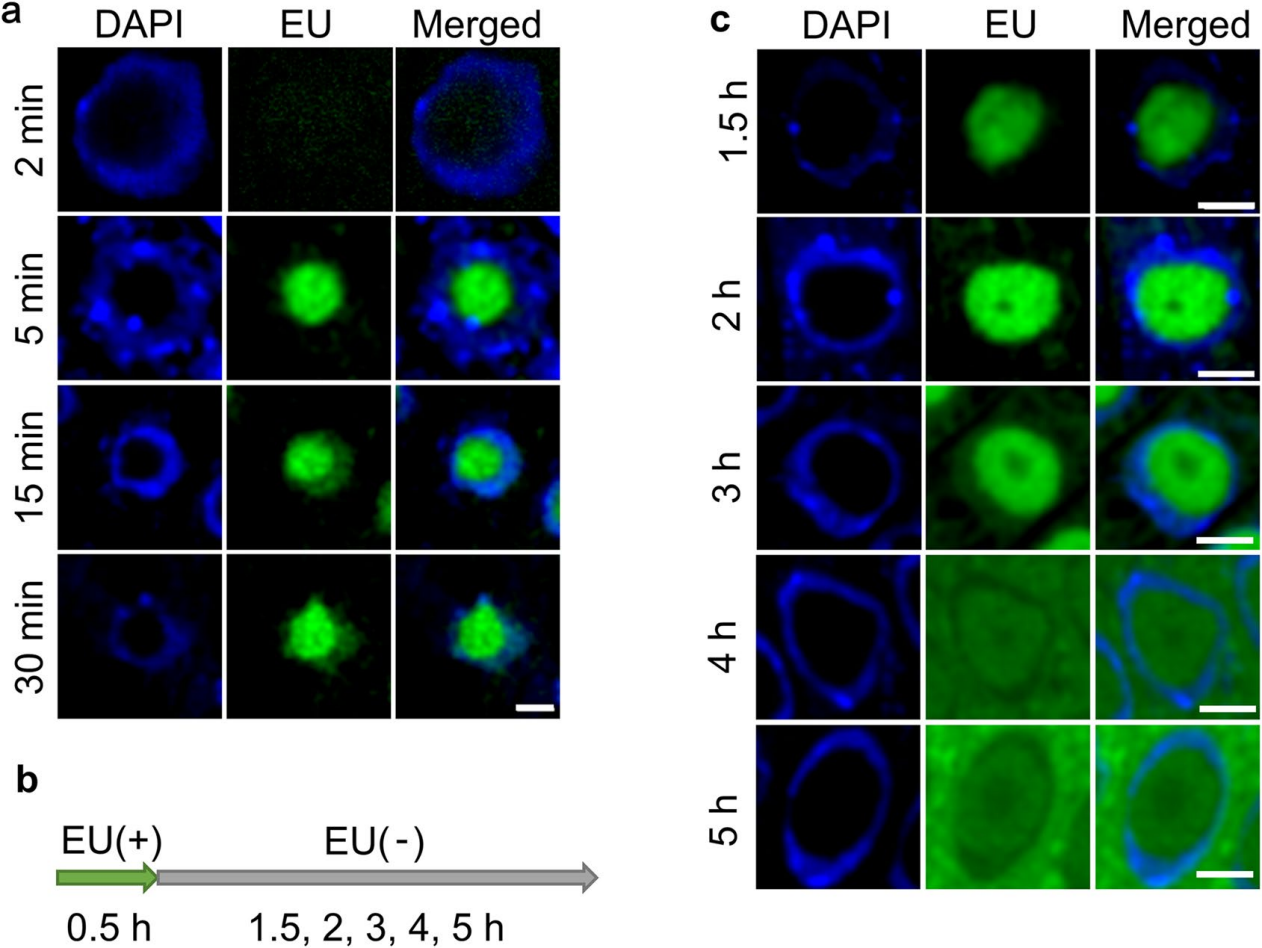
Short-term incorporation and pulse feeding of 5-ethynyl uridine (EU) into *Arabidopsis thaliana* root cells. **a** EU was incorporated into *A. thaliana* roots for 2 min, 5, 15, and 30 min. The left panels show 4′,6-diamidino-2-phenylindole (DAPI) staining, the middle panels show EU signals, and the right panels show merged images. Scale bar = 3 µm. **b** Scheme of the EU pulse-feeding experiment. **c** Results of pulse feeding at 1.5, 2, 3, 4, and 5 h after the EU pulse incorporation. The left panels show DAPI signals, the middle panels show EU signals, and the right panels show merged images. Scale bars = 3 µm

To determine the EU incorporation site in the nucleolus, EU was incorporated into *A. thaliana* roots for 2, 5, 15, and 30 min (Fig. 2a). As a result, EU was detected throughout the whole nucleolus in every experiment. The EU signals were unevenly distributed in the nucleolus. To trace the newly synthesized rRNAs, *A. thaliana* roots were incubated in medium lacking EU for 1.5, 2, 4, and 5 h after an incubation in EU-containing medium for 30 min. The scheme of these experiments is shown in Fig. 2b. As a result, EU was also detected throughout the whole nucleolus at 1.5 h after EU was released. However, EU signals were weaker at the center region of the nucleolus at the 2 and 3 h time points. Moreover, EU signals gradually weakened in the nucleolus, but increased in the cytosol region at the 4 and 5 h time points (Fig. 2c). This result indicated that EU-incorporated rRNAs were transferred from the nucleolus to the cytoplasm between 3 and 4 h after EU was released.

### EU is not incorporated into the nucleolus center region under heat and chilling stress

**Fig. 3:**
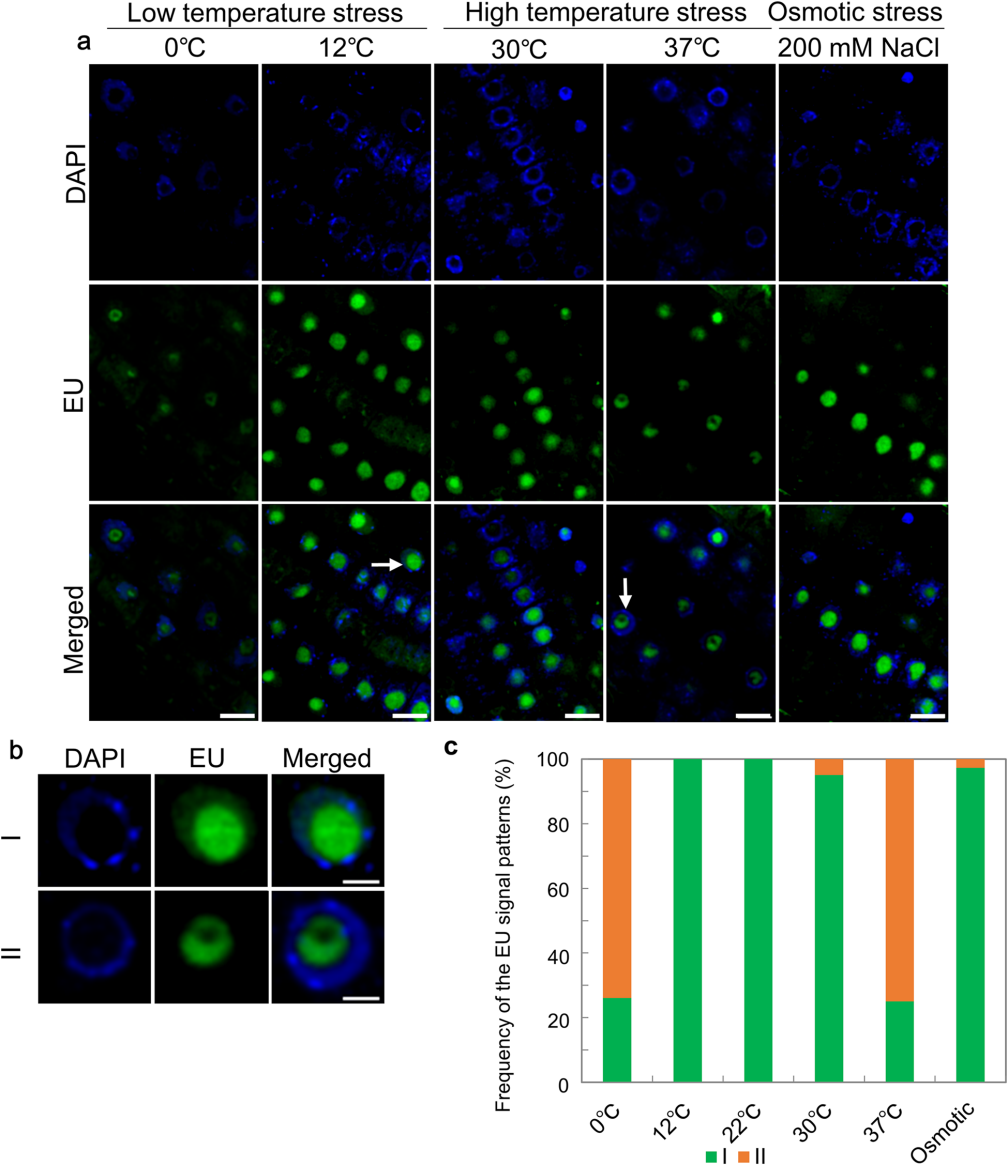
5-Ethynyl uridine (EU) incorporation under various stress treatments. **a** EU incorporation under low temperature stress (chilling stress, first left panel; cold stress, second left), high temperature stress (mild heat stress, third left panel; severe heat stress, second right panel), and osmotic stress (first right). The upper panels show 4′,6-diamidino-2-phenylindole (DAPI) staining, the middle panels show EU signals, and the lower panels show merged images. Scale bars = 10 µm. **b** EU signal patterns classified into patterns I (upper), and II (lower). Pattern I, EU distributed throughout the nucleolus (nucleus marked with arrowhead in (**a**) second panel from left); pattern II, EU not incorporated into the center region of the nucleolus (nucleus marked with arrowhead in (**a**) second panel from right). Scale bars = 3 µm. **c** Frequency of EU signal patterns under each stress treatment. *n* = 49 for control conditions, 74 for cold stress, 23 for chilling stress, 61 for mild heat stress, 28 for severe heat stress, and 37 for osmotic stress. The number of the seedlings was 3–5 per experiment

To explore the nucleolar response to various environmental stresses, we monitored the incorporation of EU under low temperature stress (chilling stress: 0 °C for 2 h, cold stress: 12 °C for 2 h), high temperature stress (mild heat stress: 30 °C for 2 h, severe heat stress: 37 °C for 2 h), and osmotic stress (200 mM NaCl, 2 h) (Fig. 3a). Following the chilling and severe heat treatments, EU was not incorporated into the center region of the nucleolus. By contrast, EU incorporation occurred similarly to that in the control under cold, mild heat, and osmotic stress. The two patterns of EU signal distribution are shown in Fig. 3b. In pattern I (Fig. 3b upper; magnifications of parts marked with an arrow in Fig. 3a second panel from left), EU was incorporated throughout the whole nucleolus. In pattern II (Fig. 3b lower; magnification of the part marked with an arrowhead in Fig. 3a third panel from right), EU was not incorporated into the center of the nucleolus. Figure 3c shows the frequency of the EU signal patterns in the stress treatments. Under chilling and severe heat stresses, about 60–80% of the nucleoli showed pattern II.

**Fig. 4:**
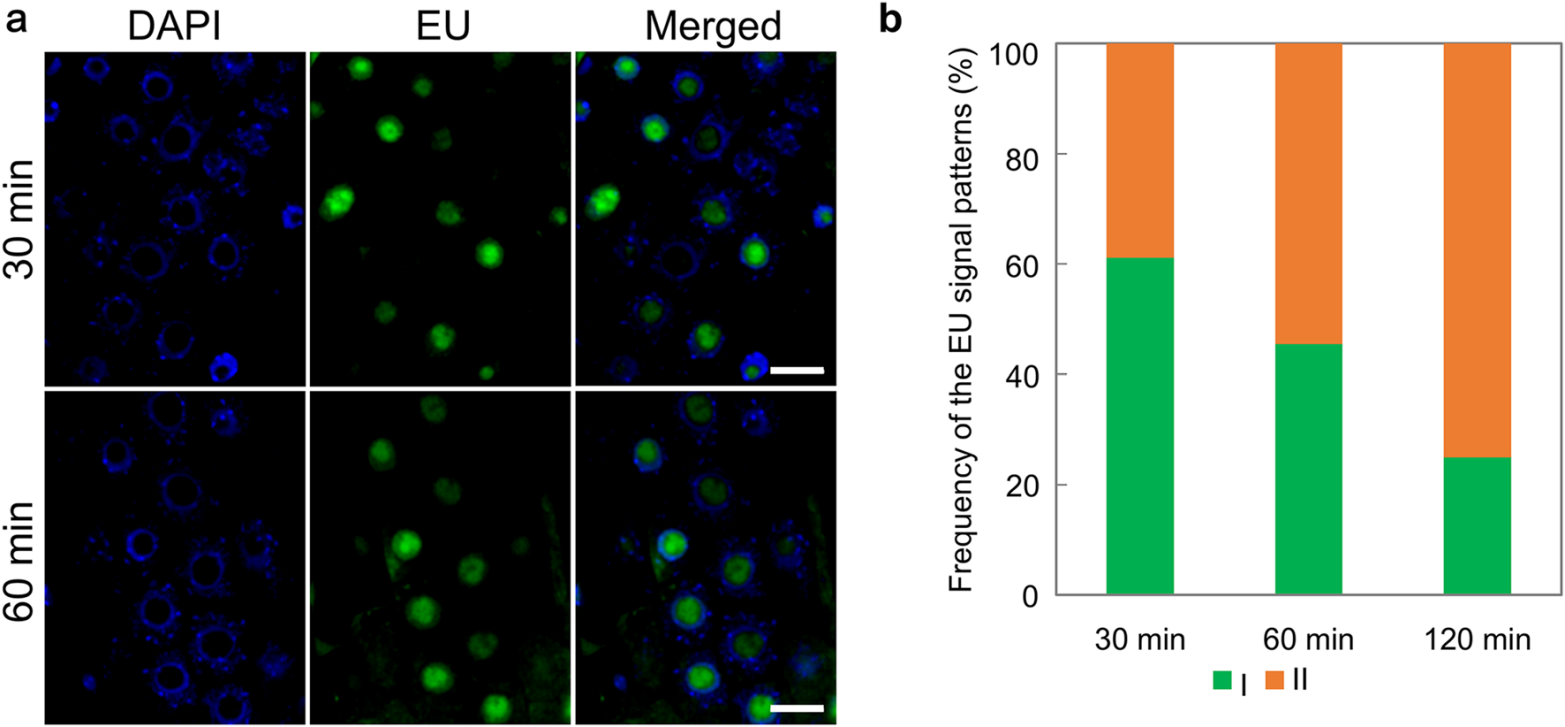
Time course analyses of EU signal patterns under severe heat stress. **a** EU incorporation under severe heat stress for 30 min (upper) and 60 min (lower). The left panels show DAPI staining, the middle panels show EU signals, and the right panels show merged images. Scale bars = 10 µmm respectively. **b** Frequency of EU signal patterns under each stress treatment. The data at 120 min is the same as shown in the data at 37 °C in Fig. 3 (**c**). *n* = 36 for 30 min, and 33 for 60 min. The number of the seedlings was 3–5 per experiment

To analyze the timing of EU incorporation pattern II, EU was incorporated for 30 and 60 min under severe heat stress (Fig. 4a, b). As a result, EU incorporation pattern II gradually increased with increasing stress duration.

### Composition of sub-nucleolus structures under heat and chilling stress

**Fig. 5:**
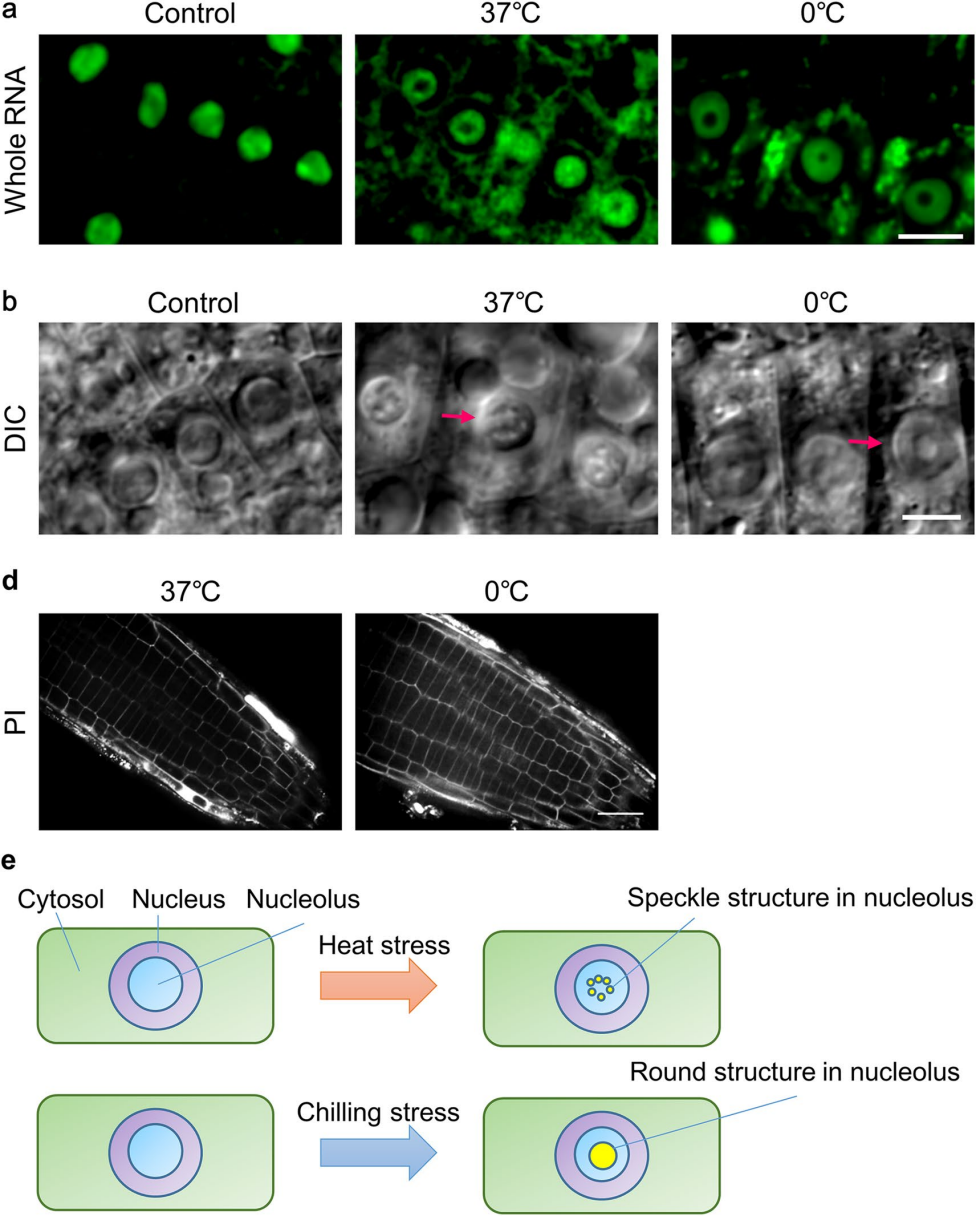
Observation of sub-nucleolus structures with Nucleolar ID and differential interference contrast imaging. **a** Nucleolar ID staining images in the control (left), heat stress treatment (middle), and chilling stress treatment (right). **b** The result of differential interference contrast. Images in the control (left), heat stress treatment (middle), and chilling stress treatment (right). Arrowheads indicate the speckled nucleolar structure in the lower middle image and round nucleolar structure in the lower right image. Scale bars = 10 µm. **c** Results of the propidium iodide staining of samples exposed to severe heat and chilling stresses. Scale bar = 50 µm. **d** Schematic of plant nucleolar dynamics under heat and chilling stresses

To analyze that the pattern composed under the chilling and severe heat stress were dependent on the EU incorporation, the rRNAs were stained with nucleolar ID (Hasegawa and Matsunaga 2014) (Fig. 5a). As shown in Fig. 5, rRNAs were not localized in the center of the nucleolus under severe heat and chilling stresses. Under these stresses, nucleolar ID signals were also localized in the cytoplasmic region. These signals may indicate the presence of a stress body constructed from mRNAs in the cytoplasm (Chantarachot and Bailey-Serres 2018).

To examine the structure in the center of the nucleolus, seedlings were observed by differential interference contrast (DIC) microscope (Fig. 5b). Under severe heat stress, speckled structures were observed in the nucleolus (Fig. 5b, arrowhead in the middle image). Under chilling stress, a round structure was observed in the center of the nucleolus (Fig. 5b, arrowhead in the right image). To assess whether the chilling and severe heat stresses resulted in irreversible damages, seedlings exposed to these stresses were stained with PI (Fig. 5c). If the seedlings were irreversibly damaged, we expected to detect PI signals in the nucleus. A lack of PI signals in the nucleus in almost all cells suggested the chilling and severe heat stresses did not irreversibly damage the seedlings. Figure 5d shows the summary of the results. These results may indicate that the observed structures inhibited the localization of the newly synthesized and whole rRNAs in the center region of the nucleolus.

The observed structures resembled the sub-nucleolus structure known as the nucleolar cavity or nucleolar vacuole found in soybean (Stępiński 2012), pea (Williams et al. 1985), and Arabidopsis (Ohbayashi et al. 2017). Previous reports showed that the nucleolar vacuole of soybean is small and gradually increases in volume during recovery from chilling stress, and that transcriptional activity increases with increasing volume of the nucleolar cavity (Stępiński 2012, 2014). In contrast to these previous reports, our results showed that nucleolar cavities were formed when chilling or heat stress was imposed. This difference may indicate differences in function between species. U1 snRNP-specific proteins and exon junction complex proteins were found in the nucleolus cavity of *A. thaliana* (Lorković and Barta 2008; Pendle et al. 2005). Conversely, according to our EU and whole-RNA staining results, RNAs were not found in the center region of the nucleolus. Thus, mRNA-related factors such as U1 snRNP and exon junction complex proteins may be collected from the nucleoplasm and stored in the nucleolar cavity to reduce transcriptional events in the nucleoplasm.
