# Correction to: Heat and chilling stress induce nucleolus morphological changes

**DOI:** 10.1007/s10265-020-01190-3

**Published:** 2020-04-25

**Authors:** Kohma Hayashi, Sachihiro Matsunaga

**Affiliations:** Department of Applied Biological Science, Faculty of Science and Technology, Tokyo University of Science, 2641 Yamazaki, Noda, Chiba 278‑8510 Japan

## Correction to: Journal of Plant Research (2019) 132:395–403 10.1007/s10265-019-01096-9

The article Heat and chilling stress induce nucleolus morphological changes, written by Kohma Hayashi and Sachihiro Matsunaga, was originally published Online First without Open Access. After publication in volume 132, issue 3, page 395–403 the author decided to opt for Open Choice and to make the article an Open Access publication. Therefore, the copyright of the article has been changed to © The Author(s) 2020 and the article is forthwith distributed under the terms of the Creative Commons Attribution 4.0 International License (https://creativecommons.org/licenses/by/4.0/), which permits use, duplication, adaptation, distribution and reproduction in any medium or format, as long as you give appropriate credit to the original author(s) and the source, provide a link to the Creative Commons license, and indicate if changes were made.

The original article was updated.

